# Qualitative Real-Time Schlieren and Shadowgraph Imaging of Human Exhaled Airflows: An Aid to Aerosol Infection Control

**DOI:** 10.1371/journal.pone.0021392

**Published:** 2011-06-22

**Authors:** Julian W. Tang, Andre D. G. Nicolle, Jovan Pantelic, Mingxiu Jiang, Chandra Sekhr, David K. W. Cheong, Kwok Wai Tham

**Affiliations:** 1 Department of Laboratory Medicine, National University Hospital, Singapore, Singapore; 2 Department of Building, School of Design and Environment, National University of Singapore, Singapore, Singapore; University of Hong Kong, Hong Kong

## Abstract

Using a newly constructed airflow imaging system, airflow patterns were visualized that were associated with common, everyday respiratory activities (e.g. breathing, talking, laughing, whistling). The effectiveness of various interventions (e.g. putting hands and tissues across the mouth and nose) to reduce the potential transmission of airborne infection, whilst coughing and sneezing, were also investigated. From the digital video footage recorded, it was seen that both coughing and sneezing are relatively poorly contained by commonly used configurations of single-handed shielding maneuvers. Only some but not all of the forward momentum of the cough and sneeze puffs are curtailed with various hand techniques, and the remaining momentum is disseminated in a large puff in the immediate vicinity of the cougher, which may still act as a nearby source of infection. The use of a tissue (in this case, 4-ply, opened and ready in the hand) proved to be surprisingly effective, though the effectiveness of this depends on the tissue remaining intact and not ripping apart. Interestingly, the use of a novel ‘coughcatcher’ device appears to be relatively effective in containing coughs and sneezes. One aspect that became evident during the experimental procedures was that the effectiveness of all of these barrier interventions is very much dependent on the speed with which the user can put them into position to cover the mouth and nose effectively.

From these qualitative schlieren and shadowgraph imaging experiments, it is clear that making some effort to contain one's cough or sneeze puffs is worthwhile. Obviously, there will be a large amount of variation between individuals in the exact hand or tissue (the most common methods) configuration used for this and other practical factors may hinder such maneuvers in daily life, for example, when carrying shopping bags or managing young children.

## Introduction

As the heightened interest in influenza continues, as a result of the recent A(H1N1) 2009 pandemic, it is useful to know what interventions are effective in preventing, or at least reducing, the transmission of infection at an individual level.

Although there have been some debates and controversies over the contribution of the airborne route to the spread of influenza [1]-[7], there is now increasing evidence that this route of transmission of influenza may play a clinically significant role in the spread of this infection.

Various teams have demonstrated the presence of influenza virus RNA in various human exhalations, including breathing [8], [9], talking [9] and coughing [9], [10], and suspended in the air in healthcare environments [11]-[13]. In addition, some studies have characterized the number and size of droplets produced by human exhalations in more detail [14], with more recent studies focusing on the amount of influenza RNA contained in droplets of different sizes [10], [12], [13].

However, the viability and survival of these airborne viruses in the environment are an important requisite for successful onward transmission [1], [15], and PCR testing to determine the presence of viral RNA alone cannot assess this. So it is significant that at least one study has demonstrated the viability of these suspended viruses showing their potential to transmit infection between individuals via the airborne route [10].

Although these studies go some way to demonstrating the initial steps of the airborne transmission pathway, i.e. source generation and environmental contamination with potentially infectious airborne droplets and droplet nuclei, further investigations into the downstream dissemination patterns of these suspended droplets is required to fully characterize the aerosol transmission risk , i.e. airflow movements generated by human exhalations and environmental activities (e.g. those induced by moving doors and people) [1], [16], [17]. Such investigations require studies using methodologies that can examine the more physical aspects (i.e. the airflow dynamics) of various human exhalations, which are a reasonable surrogate marker to delineate the potential limits of dissemination for such airborne infection [18]–[20]. These previous studies using the schlieren imaging technique mainly focused on visualizing the airflows generated by coughing, both with and without the wearing of standard surgical and N95 face masks. In this study, we have now successfully extended these qualitative airflow visualization experiments to demonstrate the exhalation flows generated by other respiratory activities (such as quiet breathing, whistling, laughing, talking and sneezing), as well as the effectiveness of specific interventions to limit the spread of potentially infectious droplets in coughs and sneezes.

## Methods

### Optical set-up

Schlieren imaging is based on the principal that light rays are refracted as they pass through media of different densities, which, in this case, is air at different temperatures. The temperature of exhaled human exhaled airflows range between 29–32°C [21] and ambient room temperatures are usually around 20–25°C. This is difference in air temperatures is sufficient to allow the visualization of human exhaled airflows using a simple optical set up which is described below.

**Figure 1:**
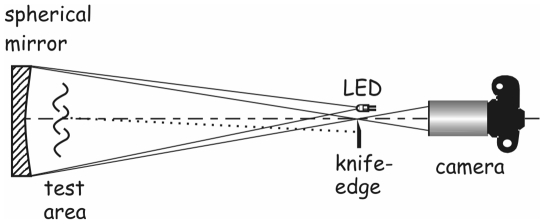
Schematic of schlieren optical imaging system set-up. Test subjects (human volunteers) typically stand about 1 meter in front and to one side (indicated by ‘test area’), and face across the mirror to maximize the reflective mirror surface within which their exhaled airflows can be visualized.

**Figure 2:**
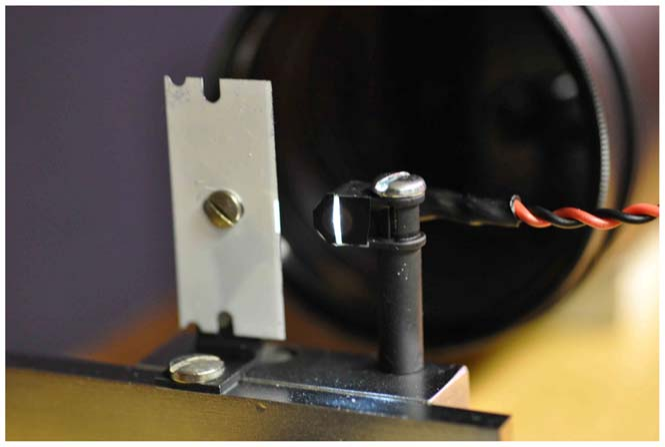
Camera and light source set-up. The LED light source is shaped into a vertical slit positioned just in front of the camera lens. When the subject is in focus, the knife edge (or razor blade painted white as shown here) is moved carefully into the path of the LED beam, cutting off part of its lateral aspect, to give the schlieren or shadowgraph effect.

The schlieren optical set-up used in these experiments consists of a large, precise (of astronomical telescope quality), 1 m-diameter, spherical, concave mirror made of fine annealed pyrex, with a 10 m radius of curvature (focal length 5 m), giving an aperture of f/5 (Cosmo optics, Inc., Middletown, NY). A diffuse, white, LED light source placed at the centre of curvature of this mirror produces a real, upright, magnified image of an experimental subject (human volunteer) when he/she is standing approximately 1 meter in front of the mirror. This image can be captured and recorded by a camera placed just behind the LED light source (Figure 1). Once the subject is in place, the image is focused in the camera viewfinder then the knife-edge (or razor-blade) is moved slowly into the field of view, cutting off part of the LED light beam to give the schlieren or shadowgraph effect (Figure 2). The extent to which the knife-edge intrudes into the LED light beam can be varied to give the most optimum airflow imaging results, depending on the respiratory activity being performed.

**Figure 3:**
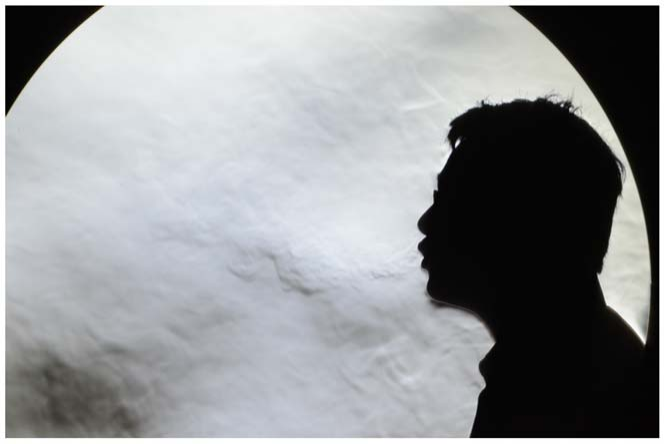
Typical position of a single subject in front of the schlieren mirror. The exhalation puffs can be seen as disturbances in the ‘texture’ in front of the head (images obtained using a Nikon D7000 SLR in HD 1920×1080 live video mode).

**Figure 4:**
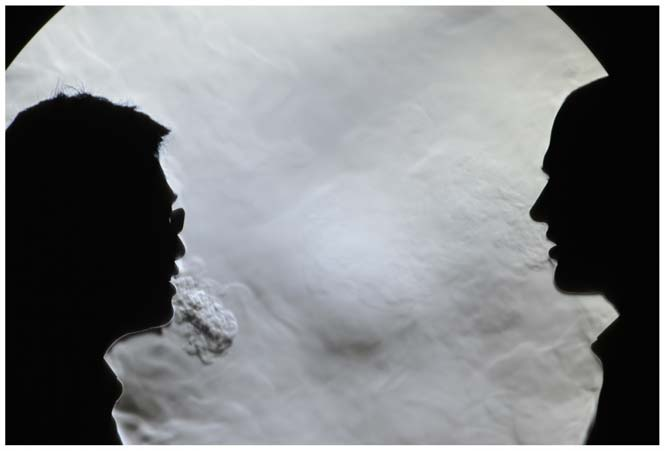
Two subjects in position in front of the schlieren mirror. For some experiments, two subjects may face each other across the mirror in order to visualize how their exhaled airflows interact during, e.g. during normal conversation (images obtained using a Nikon D7000 SLR in HD 1920×1080 live video mode).

Typically, for most exhalation experiments, the human subject will stand to one side of the mirror, facing across its surface to enable as much of his/her exhalation puff to be visualized in the 1 m-diameter surface area of the mirror as possible (Figure 3). In some experiments (e.g. to visualize airflow patterns during a conversation between two people), it is also possible for two subjects to stand facing each other across the mirror (Figure 4).

### Human volunteer experiments

#### Ethics statement

All experiments in this study involving human volunteers were approved by the Domain Specific Review Board of the National University Hospital/ National University Health System (DSRB ref no. E/09/024). All volunteers participating in this study gave both their written and verbal consent.

One of the main advantages with this schlieren imaging system is that there are no additional, potentially irritant or toxic tracer particles or gases required for airflow visualization. The subject simply has to stand in front of the mirror and exhale normally. The human respiratory airflow behaviors that were investigated in this study included: i) coughing and ii) sneezing, with various forms of interventions, i.e. using hands, tissues and a novel ‘coughcatcher’ device (available from: http://www.coughcatcher.com/) to contain the cough and sneeze puffs. This is a sort of modified mask that can be carried, folded flat in one's pocket and opened to cover the nose and mouth when a person is about to cough (or sneeze). The effectiveness of wearing surgical and N95 masks was also tested. To induce sneezing, the human volunteer subject just sniffed a little ground black pepper to stimulate the sneeze reflex, just prior to triggering the camera for recording. Additional images were captured of airflow patterns generated by iii) nose and mouth breathing, as well as, iv) whistling and v) laughing, and vi) talking, with two people in conversation, to demonstrate the nature of the exhalation flows generated by these activities.

For these series of qualitative images a variety of black-and-white schlieren and shadowgraph (a form of higher contrast, de-focused ‘schlieren’) images were captured as still images (at a resolution of up to 16 megapixels per image) or real-time video in high definition (HD) at 1920×1080 pixel resolution at 24 frames per second (Nikon D7000 SLR, Nikon Inc., Melville, NY), or a series of TIF images at 1024×1024 pixel resolution at a frame-rate of 500–5000 frames per second (fps) using a high-speed camera (Photron SA1.1 camera, Dynamic Analysis System, Pte Ltd, Singapore). Video editing and annotating was performed using a combination of Corel VideoStudio Pro X3 (Corel Corp., Ottawa, Canada), and QuickTime Video Converter (4VideoSoft Studio, v.3.3.12, http://www.4videosoft.com/index.html) and Windows Movie Maker (v.5.1., Microsoft Corp., Redmond, WA, USA).

## Results

A series of high contrast, digital black-and-white schlieren and shadowgraph images of everyday respiratory activities were captured. The results are in the form of annotated video clips as these are much more illustrative for this technique - still images do not display the dynamic nature of the airflow patterns adequately.

i) coughing (video S1, Photron SA1.1, 1000 fps, 1024×1024 pixel resolution, playback at half-speed, with a 32-year old male subject): this annotated series of shadowgraph video clips (displayed in slow-motion at half-speed for clarity) shows a natural, uncovered cough as a control, followed by a series of interventions that people may perform in everyday life, i.e. putting a fist or open hand or tissue across the nose and mouth. Airflow patterns demonstrating the effectiveness of novel ‘coughcatcher’ device and the surgical and N95 masks are also shown. It was found that the effectiveness of any of these interventions depends on the speed and the configuration with which the hand or tissue is applied. With the tissue, the thickness was also important (i.e. very thin tissues, e.g. 2-ply, may just rip). None of these interventions completely contained the escaping puffs. Although escaping airflows were visible passing through, above and around the sides of these barrier interventions, significant portions of these airflows were blocked, decelerated and/or redirected into the convective, upward-rising, body thermal plume, resulting in far less of the exhaled air traveling forward into the potential breathing zone of any nearby person.

ii) sneezing (video S2, Photron SA1.1, 3000 fps, 1024×1024 pixel resolution, playback at half-speed, with a 32-year old male subject): similar to the cough series above, this annotated series of shadowgraph video clips (again displayed in slow-motion at half-speed for clarity) shows a natural, uncovered sneeze as a control, followed by the same series of everyday interventions as was used with coughing. Similar findings about the ability to curtail the dissemination of the sneezed droplets by properly covering the nose and mouth quickly enough with the hand, tissue or ‘coughcatcher’ device, also applied to sneezing. Putting these interventions into place in time was noticeably more problematic than with coughing, as the sneezing reflex was more difficult to control and sometimes it was not possible to ‘hold’ the sneeze for long enough to position the intervention correctly for optimum containment - as is often the case in everyday life. The effectiveness of surgical and N95 masks in containing the sneeze puff is also shown here. As for the cough, although none of these interventions completely contained the sneeze puff, they all blocked, decelerated and redirected portions of the puff into the upward-rising body thermal plume to some extent, which would limit the exposure to any individual standing nearby.

iii)-v) nasal/mouth-breathing, whistling, laughing, coughing (video S3, Photron SA1.1, 500 fps, 1024×1024 pixel resolution, playback in real-time, with a 32-year old male subject): this annotated series of shadowgraph video clips (displayed in real-time) shows a series of respiratory activities that are performed everyday by many people. The difference in potential for nasal- and mouth-breathing to transmit infection can be seen with the conical nasal-breathing puffs being mainly directed downwards, away from another person's breathing zone, unless they are either seated (e.g. students in a classroom) or very short (e.g. children). Mouth-breathing directs a puff more horizontally and potentially directly into the face of an individual standing opposite, e.g. during a conversation. Similarly, the exhalation puffs produced by whistling and particularly laughing are also directed mainly horizontally, again with the potential for contaminating the breathing zone of other people nearby. Additional video footage of coughing (i.e. natural and with surgical and N95 masks) has also been included in this clip, shown in real-time, for comparison with the slow-motion footage shown in video S1.

vi) talking (video S4, Nikon D7000 SLR, 24 fps, 1920×1080 pixel resolution, playback in real-time, with two male subjects ages 42 and 30 years): this video clip of two male subjects talking, situated on opposing edges of the mirror (i.e. about 1 m apart), demonstrates the typical exhalation airflows that one might be exposed to during a conversation between two people. Note that most of the time, the puffs remain separate, though they do overlap and interact occasionally and enter each others' breathing zones. Also note that the visible exhaled puffs from the subject on the left are often a combination of puffs emanating from the nose and mouth, which occur far less frequently from the subject on the right, demonstrating that exhalation airflow patterns may vary widely amongst individuals.

## Discussion

Previous studies on the visualization of airflow patterns have used techniques involving the use of tracer particles or gas with laser light illumination [22]-[27]. As these methods are hazardous to humans, they have been applied to various types of manikins rather than human volunteers. Admittedly, some of these manikins are quite sophisticated, with the ability to accurately simulate human skin temperatures and the resultant human thermal plume [28].

Whilst the conclusion from these models have been useful, there is always the remaining question of how these airflows behave when generated by actual human volunteers. Airflow patterns of coughing human volunteers of different ages, with and without the wearing of various face masks, have been investigated elsewhere using this schlieren principle with a large 1 m-diameter, modified, parabolic mirror [18]–[20]. However, this present study is the first description of a modern, large, spherical, concave mirror, specifically designed and constructed for this specific purpose of visualizing exhaled human airflows.

Regarding everyday interventions for coughing and sneezing, there is a great deal of individual variation on how quickly and in what configuration these hand positions are applied to the nose and mouth, with a subsequent variation in the degree of effectiveness in containing these exhaled puffs. Similarly, where a tissue is used, its effectiveness in containing the cough or sneeze puffs will depend on its thickness and the degree with which it can be opened and placed over the nose and mouth in time. The novel ‘coughcatcher’ device appears to be quite effective in containing the cough and sneeze puffs, but this also requires quick opening and accurate placement – and its use maybe more costly and generate more waste (as it contains plastic) than the other interventions described here. Although there appears to be some airflow escaping through the front of the surgical and N95 masks, this may not necessarily pose an infectious risk due to the various mechanical and electrostatic filtering mechanisms present in these masks. However, it is clear that when compared to the natural (i.e. unobstructed) cough and sneeze puffs, some intervention is better than none – especially in crowded areas, such as on public transport or other public venues.

Recent recommendations from the US Centers for Disease Control and Prevention to cover one's mouth or nose with an elbow or sleeve (http://www.cdc.gov/flu/protect/covercough.htm) have not been specifically investigated in these series of images, but this is planned in the near future. However, it should be noted that the act of coughing or sneezing into one's sleeve may not be personally or socially acceptable to all people in different cultures.

Although not specifically investigated here, singing is another common respiratory activity, where the mouth is rarely deliberately covered, which may produce similar horizontal puffs with the potential risk for the transmission of infection. The dissemination potential of operatic versus popular singing may also differ [29]. In addition, singers often perform facing out to large, captive audiences for several hours.

Typical conversation at distance of the order of 1 m apart appears to be safe for much of the time, but it can be seen that individuals talk in very different ways with a large variety of airflows patterns, even when speaking the same words. Such variation may also occur within the same individual depending on different situations, e.g. relaxed conversation versus a heated argument. Hence differences in pronunciation and elocution, as well as talking in different languages [30], may all be significant variables affecting the generation of potentially infectious aerosols during human speech.

Although computational algorithms are being developed to analyze these real-time images more quantitatively in 2-D with a potential to extrapolate these results to 3-D, this may only be useful as an academic exercise as, given the immense variety of these respiratory behaviors (even within a single person), the actual clinical utility of this data may be quite limited. A similar comparison with hand washing can be made. Although studies have been performed to quantify the number of bacteria on hands for various reasons, e.g. to test the effectiveness of a hand cleanser on specific bacteria [31]–[35], for everyday hand hygiene and infection control purposes, it is not necessary to know how many bacteria are on our hands in order to understand the need for hand-washing as an infection control measure. In this light, these qualitative schlieren images certainly confirm the effectiveness of masking a human source of infectious aerosols, whilst they are coughing and sneezing, to reduce the dissemination of infectious agents. In addition, almost no leakage is seen (images not shown) from the exhaled puffs produced from the other respiratory activities investigated (i.e. breathing, talking, whistling and laughing). These findings support existing recommendations to ‘mask the patient’ where possible to limit the spread of airborne infections such as avian A(H5N1) and pandemic A(H1N1) influenza [36]–[39].

Hence, this newly constructed schlieren optical imaging system allows human volunteer subjects to behave in a natural manner in front of the imaging mirror, without the need for any potentially irritant or toxic tracer substances, or any hazardous (e.g. laser) light source. Whilst these schlieren and shadowgraph video images are essentially qualitative, they do effectively illustrate the variety of behavior of human exhaled airflows produced by various everyday respiratory activities.

Understanding the dynamics of these human exhaled airflows is only part of the larger puzzle in determining their potential to disseminate airborne infection. These findings will need to be taken together with those from other ongoing studies in droplet (as opposed to airflow) dynamics and investigations into the typical concentrations and viability of suspended viruses produced by the respiratory activities of infected individuals, in order to determine the true risk of disseminating aerosolized and airborne infection to others.

However, despite this, it is of note that routine checks at the point of air entry for the effectiveness and maintenance of clinical and laboratory aerosol biocontainment facilities currently only rely on airflow (i.e. non-droplet-related) parameters, such as negative pressure gradients across doorways in hospital isolation rooms [40], and minimum inflow air velocities across the front sash opening for laboratory biosafety cabinets [41].

## Supporting Information

Video S1 **Coughing.** This annotated series of shadowgraph video clips (displayed in slow-motion at half-speed for clarity) shows a natural, uncovered cough as a control, followed by a series of interventions that people may perform in everyday life, i.e. putting a fist or open hand or tissue over the nose and mouth, as well as the effectiveness of a novel ‘coughcatcher’ device, a surgical and N95 mask in containing the cough puff (also referred to as ‘plumes’ in the video). Images captured using the Photron SA1.1, at 1000 fps, at 1024×1024 pixel resolution, formatted for playback at half-speed, with a 32-year old male subject.
(WMV)

Video S2 **Sneezing.** This annotated series of shadowgraph video clips (displayed in slow-motion at half-speed for clarity) shows a natural, uncovered sneeze as a control, followed by a series of interventions that people may perform in everyday life, i.e. putting a fist or open hand or tissue over the nose and mouth, as well as the effectiveness of a novel ‘coughcatcher’ device, a surgical and N95 mask in containing the sneeze puff (also referred to as ‘plumes’ in the video). Images captured using the Photron SA1.1, at 3000 fps, at 1024×1024 pixel resolution, formatted for playback at half-speed, with a 32-year old male subject.
(WMV)

Video S3 **Nasal/mouth-breathing, whistling, laughing, coughing.** This annotated series of shadowgraph video clips (displayed in real-time) shows a series of these everyday respiratory activities. The coughing footage (natural and with surgical and N95 masks) is shown again here, but in real-time. Images captured using the Photron SA1.1, at 500 fps, at 1024×1024 pixel resolution, formatted for playback in real-time, with a 32-year old male subject).
(WMV)

Video S4 **Talking.** This video clip of two male subjects talking, situated on opposing edges of the mirror (i.e. about 1 m apart), demonstrates the typical exhalation flows that one might be exposed to during a conversation between two people. Images captured using the Nikon D7000 SLR, at 24 fps, at 1920×1080 pixel resolution, formatted for playback in real-time, with two male subjects ages 42 and 30 years.
(WMV)
